# Retraction Note: Solar energy optimization in solar-HVAC using Sutterby hybrid nanofluid with Smoluchowski temperature conditions: a solar thermal application

**DOI:** 10.1038/s41598-023-34628-4

**Published:** 2023-05-09

**Authors:** Wasim Jamshed, Mohamed R. Eid, Rabia Safdar, Amjad Ali Pasha, Siti Suzilliana Putri Mohamed Isa, Mohammad Adil, Zulfiqar Rehman, Wajaree Weera

**Affiliations:** 1 Department of Mathematics, Capital University of Science and Technology (CUST), Islamabad, 44000 Pakistan; 2 Department of Mathematics, Faculty of Science, New Valley University, Al‑Kharga, Al‑Wadi Al‑Gadid, 72511 Egypt; 3 Department of Mathematics, Faculty of Science, Northern Border University, Arar, 1321 Saudi Arabia; 4 Department of Mathematics, Lahore College Women University, Lahore, Pakistan; 5 Aerospace Engineering Department, King Abdulaziz University, Jeddah, 21589 Saudi Arabia; 6 Centre of Foundation Studies for Agricultural Science, Universiti Putra Malaysia, Seri Kembangan, Malaysia; 7 Mechanical Engineering Program, Physical Science and Engineering Division, King Abdullah University of Science and Technology, Thuwal, 23955‑6900 Saudi Arabia; 8 KAUST Clean Combustion Research Center, King Abdullah University of Science and Technology, Thuwal, 23955‑6900 Saudi Arabia; 9 Department of Mathematics, Air University, Islamabad, 44000 Pakistan; 10 Department of Mathematics, Faculty of Science, Khon Kaen University, Khon Kaen, 40002 Thailand

Retraction of: *Scientific Reports* 10.1038/s41598-022-15685-7, published online 07 July 2022

The Editors have retracted this Article.

After publication concerns were raised about the use of nonsensical language and excessive citation of work that is not directly relevant to the subject of this article. The Editors requested an explanation from the Authors; however, their response did not adequately address these concerns. The Editors therefore no longer have confidence in the reliability of the conclusions presented.

Wasim Jamshed, Mohamed R. Eid, Rabia Safdar, Amjad Ali Pasha, Siti Suzilliana Putri Mohamed Isa, Mohammad Adil and Wajaree Weera disagree with this retraction. Zulfiqar Rehman has not responded to correspondence from the Editors about this retraction.

